# A Phase I/II study of lomustine and temozolomide in patients with cerebral metastases from malignant melanoma

**DOI:** 10.1038/sj.bjc.6603503

**Published:** 2006-12-05

**Authors:** J M G Larkin, S A Hughes, D A Beirne, P M Patel, I M Gibbens, S C Bate, K Thomas, T G Eisen, M E Gore

**Affiliations:** 1 Department of Medicine, Royal Marsden Hospital, London SW3 6JJ, UK; 2 Cancer Research UK Clinical Centre, St James University Hospital, Beckett Street, Leeds LS9 7TF, UK; 3 Academic Division of Clinical Oncology, Nottingham University, City Campus, Nottingham NG5 IPB, UK

**Keywords:** cerebral metastases, melanoma, chemotherapy, lomustine, temozolomide

## Abstract

Temozolomide is an alkylating agent with activity in the treatment of melanoma metastatic to the brain. Lomustine is a nitrosurea that crosses the blood brain barrier and there is evidence to suggest that temozolomide may reverse resistance to lomustine. A multicentre phase I/II study was conducted to assess the maximum-tolerated dose (MTD), safety and efficacy of the combination of temozolomide and lomustine in melanoma metastatic to the brain. Increasing doses of temozolomide and lomustine were administered in phase I of the study to determine the MTD. Patients were treated at the MTD in phase II of the study to six cycles, disease progression or unacceptable toxicity. Twenty-six patients were enrolled in the study. In phase I of the study, the MTD was defined as temozolomide 150 mg m^−2^ days 1–5 every 28 days and lomustine 60 mg m^–2^ on day 5 every 56 days. Dose-limiting neutropaenia and thrombocytopaenia were observed at higher doses. Twenty patients were treated at this dose in phase II of the study. No responses to therapy were observed. Median survival from starting chemotherapy was 2 months. The combination of temozolomide and lomustine in patients with brain metastases from melanoma does not demonstrate activity. The further evaluation of this combination therefore is not warranted.

Metastatic melanoma generally has a poor prognosis and few effective systemic treatment options. The median survival for patients with this disease is 6–9 months although 10–20% of patients will survive for 5 years (Balch *et al*, 2001). The intravenously administered alkylating agent dacarbazine (DTIC) is a standard treatment for metastatic melanoma with single agent response rates reported in the range of 5–15% (Falkson *et al*, 1998; Chapman *et al*, 1999; Middleton *et al*, 2000; Avril *et al*, 2004). The brain is a common site of metastasis in melanoma and the standard treatment for brain metastases is radiotherapy although surgery has a role in selected patients. Dacarbazine is generally ineffective at treating brain metastases from melanoma although a response rate of 7% with a further 29% of patients experiencing disease stabilisation has been reported with the related orally administered alkylating agent temozolomide as a single agent (Agarwala *et al*, 2004). Temozolomide is associated with a response rate of 13–25% in the treatment of metastatic melanoma outside the brain and is generally well tolerated with myelosuppression the major toxicity (Bleehen *et al*, 1995; Middleton *et al*, 2000; Bafaloukos *et al*, 2005; Kaufmann *et al*, 2005).

Lomustine (CCNU) is an orally administered nitrosurea that crosses the blood brain barrier and has been used in combination chemotherapy regimens such as ‘BOLD’ (bleomycin, vincristine, lomustine, dacarbazine) in metastatic melanoma (Vuoristo *et al*, 1994; Punt *et al*, 1997; Vuoristo *et al*, 2005). Trials of a related compound, fotemustine, have reported response rates of 5–25% in melanoma metastatic to the brain (Jacquillat *et al*, 1990; Mornex *et al*, 2003; Avril *et al*, 2004) and fotemustine has also been given in combination with dacarbazine (Avril *et al*, 1990; Merimsky *et al*, 1992; Lee *et al*, 1993; Chang *et al*, 1994; Comella *et al*, 1997; Richard *et al*, 1998; Seeber *et al*, 1998) and with temozolomide (Marzolini *et al*, 1998; Gander *et al*, 1999). These drug combinations are generally well tolerated although unexpected pulmonary toxicity was reported in two studies of the combination of dacarbazine and fotemustine (Gerard *et al*, 1993; Lee *et al*, 1993). Pulmonary toxicity has also been reported in two other combination studies of alkylating agents in melanoma: two of 17 patients in a study of carmustine and streptozocin developed fatal pulmonary toxicity (Smith *et al*, 1996) while three of 23 patients in a phase II trial of the BOLD regiment developed pneumonitis (Nathan *et al*, 1997).

The primary mechanism of action of lomustine is the alkylation of the O^6^ position of guanine-containing bases in DNA and the enzyme O^6^-alkylguanine transferase mediates *in vitro* resistance to both lomustine and temozolomide (Baer *et al*, 1993). Temozolomide has been shown to deplete O^6^-alkylguanine transferase both *in vitro* (Lacal *et al*, 1996) and *in vivo* (Gander *et al*, 1999) and there is a rationale, therefore, for combining lomustine and temozolomide in melanoma metastatic to the brain. It should be noted that if the predominant *in vivo* mechanism of temozolomide resistance was mediated via O^6^-alkylguanine transferase, the repeated administration of temozolomide might be expected to reverse resistance to its own activity, but this has not been observed in the treatment of melanoma. There are no clinical data reported for pharmacokinetic interaction between temozolomide and lomustine although there is no interaction between temozolomide and fotemustine (Marzolini *et al*, 1998).

Given the modest but definite active of temozolomide in the treatment of brain metastases from melanoma and the possibility of synergy between temozolomide and lomustine, a multicentre phase I/II trial was conducted to determine the maximum tolerated dose (MTD), safety and efficacy of the combination of these drugs.

## PATIENTS AND METHODS

### Patients

Patients were eligible for enrolment if they had histologically confirmed primary or secondary malignant melanoma with radiological evidence of brain metastases. In the phase II section of the study, eligible patients had bi-dimensionally measurable brain metastases of at least 2 cm in size on either contrast-enhanced CT or MRI. Previous systemic therapy was permitted for the treatment of melanoma and patients must have recovered from the effects of major surgery. Steroid therapy was permitted provided the dose was stable for at least 1 week before the administration of the trial treatment. Other inclusion criteria were: age of at least 18 years, WHO performance status of 0 to 2, life expectancy of 12 weeks or greater, adequate bone marrow function (haemoglobin >10 g dl^–1^, absolute neutrophil count >1.5 × 10^9^ l^–1^ and platelet count >100 × 10^9^ l^−1^), adequate renal function (plasma creatinine <120 *μ*mol l^–1^ or calculated creatinine clearance >50 ml min^–1^ and urea less than twice the laboratory upper limit of normal (ULN)) and adequate hepatic function (total and direct serum bilirubin <1.5 times laboratory ULN, alanine aminotransferase , aspartate aminotransferase and alkaline phosphatase <2 times the laboratory ULN). All patients provided written informed consent.

Exclusion criteria were systemic anticancer therapy within 4 weeks of study entry, prior fotemustine, lomustine or temozolomide therapy, prior whole-brain irradiation, radiation therapy given to 30% or more of the bone marrow, unresolved toxicities from previous therapies, acute infection or other uncontrolled medical co-morbidity, inability to take oral medication, poor respiratory reserve due to either a large volume of pulmonary metastases or coexisting medical conditions, previous or concurrent malignancies at other sites with the exception of surgically treated carcinoma-*in-situ* of the uterine cervix and basal or squamous cell carcinoma of the skin, pregnant or breastfeeding females and potentially fertile subjects not using effective contraception.

### Study design and statistical methods

The trial recruited at three centres in Leeds and London, UK from July 2000 to March 2003. Approval for the study was obtained from the local Ethics Committees. In phase I of the trial, a standard design was adopted. The first patient was entered at dose level 1 and observed for 4 weeks. When no acute, severe or irreversible toxicity was observed, two more patients were entered at dose level 1 at weekly intervals. If no dose-limiting toxicity (DLT) was observed, the next patient was recruited to dose level 2 when the first patient completed two cycles of treatment. If grade 3 or 4 toxicity other than alopecia or inadequately treated nausea or vomiting was observed in two out of three patients, the dose level below was expanded to six patients. The MTD was defined as the dose level below the level at which two of three patients experienced DLT, provided no more than two of the six experienced DLT at that level. For phase II of the study, a Gehan 2 stage design (Gehan, 1961) was used. To detect a response rate of 15% with a probability of a type I error of 5% and a type II error of 10% required the enrolment of 21 patients in stage 1. In the event that one or more responses were observed in stage 1, accrual would continue with a boundary condition that at least five responses had occurred by the time that 62 patients had been enroled. The primary end point of phase I was the MTD of temozolomide and lomustine and of phase II the response rate at the phase I MTD. Secondary end points included progression-free and overall survival. Statistical analyses for baseline demographics, response rates and adverse events were descriptive and Kaplan–Meier analysis was used for survival data.

### Treatment

Temozolomide was administered orally on days 1–5 of a 28-day cycle. Lomustine was administered orally on day 5 every 56 days,that is, on every alternate cycle of temozolomide. The doses of temozolomide and lomustine were escalated in phase I of the study as shown in Table 1. In phase II of the study, the dose administered was the MTD as defined in phase I, subject to dose modification in the event of toxicity as described below.

### Dose modifications

Clinical toxicity was evaluated before the start of each cycle of therapy and nadir haematological toxicity measured by full blood count at days 15 and 21 of each cycle. Lung function testing was performed at baseline and subsequently in the event of cough or dyspnoea not explained by intercurrent illness for example, pneumonia. Treatment was then withheld until interstitial pneumonitis was excluded. Treatment was administered on day 1 of a cycle if the absolute neutrophil count was at least 1.5 × 10^9^ l^–1^ and the platelet count at least 100 × 10^9^ l^–1^. In the event that the blood counts were below these thresholds, treatment was not given and the blood count repeated after a week. If blood counts were sufficient after a 1-week delay, treatment was continued as planned but if a 2-week delay occurred, treatment was administered at one dose level lower. In the event that blood counts had not recovered after 2 weeks, treatment was stopped and DLT assumed. Dose-limiting toxicity was also assumed in the event of grade 4 thrombocytopaenia of any duration, the need for platelet transfusion, nadir neutropaenia for greater than 7 days or an episode of febrile neutropaenia.

### Toxicity and response assessments

Response assessments were made according to the WHO criteria. Patients were evaluated for clinical response at the end of each cycle of therapy and in the absence of evidence of progression or significant toxicity, staging CT or MRI was repeated after the third and sixth cycles of treatment. Treatment was discontinued in the event of clinical or radiographic evidence of progressive disease and the patient assessed for radiotherapy. In the absence of disease progression and significant toxicity, six courses of therapy were administered and the patient was assessed for radiotherapy.

## RESULTS

Baseline patient characteristics are shown in Table 2. Median time from the diagnosis of primary melanoma to brain metastases was 36 months (range 1–252 months) and median time from diagnosis of brain metastases to trial entry was 1 month (range 1–8 months). No DLTs occurred in the three patients treated at dose level 1 after two cycles of therapy (Table 1). The first patient treated at dose level 2 was admitted to hospital on day 26 of cycle 1 with a fever, grade 3 anaemia, grade 4 neutropaenia and grade 4 thrombocytopaenia. The patient recovered after treatment with antibiotics, intravenous fluids, GCSF and blood products. The patient was noted to be dyspnoeic; grade 3 pulmonary fibrosis was diagnosed and the patient was withdrawn from the trial. No DLT was noted in the next two patients recruited to dose level 2. The first patient treated at dose level 3 had grade 3 thrombocytopaenia after cycle 1 resulting in a 2-week delay in administering cycle 2 of therapy but experienced no other DLT. The next two patients recruited at this dose level both developed grade 4 thrombocytopaenia and grade 4 neutropaenia. One of these patients bled into a brain metastasis and subsequently died as a result of sepsis.

As a result of the DLT observed at dose level 3, dose level 2 was expanded and three further patients were treated. Dose-limiting toxicity in the form of grade 4 thrombocytopaenia was observed in one of these patients and as a result the MTD was defined, therefore, at dose level 2, that is, temozolomide 150 mg m^–2^ days 1–5 every 28 days and lomustine 60 mg m^–2^ on day 5 every 56 days.

Fourteen patients were subsequently treated at dose level 2 in phase II of the study. For the purpose of the analyses of safety and efficacy, data from these patients were combined with the six patients treated at dose level 2 in phase I of the study to give a total of 20 patients. The majority of patients (65%) received only one cycle of therapy and only 10% of patients received more than two cycles of therapy (Table 3). No responses to therapy were seen (Table 4) although one patient received six cycles of therapy with stable disease on brain imaging. The median time from starting chemotherapy to death was 2 months with only two patients alive at 1 year and one alive at last follow-up (58 months). Of the 13 patients that only received one cycle of therapy, two had progressive disease outside the brain while five had progressive disease within the brain; all were withdrawn from the study. The remaining six patients were withdrawn from the study due to toxicities arising as a result of treatment (Table 5). Three of these patients died. Two patients with grade 4 thrombocytopaenia died with intracerebral bleeding and a further patient died as a result of pneumonia with grade 4 neutropaenia.

Myelosuppression was the major significant toxicity recorded: 30% of patients had grade 3 or 4 neutropaenia and 35% had grade 3 or 4 thrombocytopaenia (Table 5). Grade 3 or 4 haemorrhage occurred in 20% of patients while 10% of patients had grade 3 or 4 sepsis. Grade 3 or 4 anaemia was reported in 10% of patients. Half of patients reported mild or moderate fatigue and approximately a third of patients had grade 1 or 2 constipation, nausea or headache. One patient was diagnosed with pulmonary fibrosis as described above.

## DISCUSSION

The maximum-tolerated dose of temozolomide and lomustine was defined in phase I of this study as temozolomide 150 mg m^–2^ given on days 1–5 every 28 days and lomustine 60 mg m^–2^ given on day 5 every 56 days. The DLTs of a dose of 80 mg m^–2^ of lomustine in combination with 150 mg m^–2^ of temozolomide were thrombocytopaenia and neutropaenia and even at 60 mg m^–2^ of lomustine 50% of patients experienced at least grade 2 thrombocytopaenia. There have been no other reports of temozolomide administered according to the standard 5 day schedule in combination with lomustine. Since this study was completed, it has been reported that in patients with malignant high grade glioma, temozolomide can be administered at a dose of 75 mg m^–2^ per day continuously for 28 days in combination with lomustine at a dose of 100 mg m^–2^ on day 1 every 56 days without excessive toxicity (Tafuto *et al*, 2006). It should be noted, however, that extended schedule temozolomide administration is not standard therapy for metastatic melanoma although an extended temozolomide schedule is currently under investigation in a phase III study in metastatic melanoma in comparison with dacarbazine (EORTC protocol 18032).

Twenty patients were treated in this study at the MTD of temozolomide and lomustine. No responses to treatment were observed and although only seven patients received two or more cycles of therapy, it is unlikely that the combination of lomustine and temozolomide has significant activity in melanoma with brain metastases. Progressive disease (both within and outside the brain) was the most common reason for discontinuing therapy after only one cycle. This highlights both the difficulty of conducting clinical trials in this patient group and the need for effective treatment agents with a rapid onset of action. An important observation in this study is that the median survival from starting chemotherapy was 2 months, which emphasises the very poor prognosis of patients with brain metastases from melanoma, even with good performance status. This figure is similar to the median overall survival of 3.2 months reported in the largest clinical trial of patients (*N*=151) with brain metastases from melanoma reported to date (Agarwala *et al*, 2004). It could be argued that restaging patients after three cycles of treatment (as opposed to two cycles) in the study reported here may have underestimated the response rate, but this would be true only for very short-lived responses, which would be of questionable clinical significance. It is also possible that the requirement that CNS lesions be at least 2 cm in size in this study may have predisposed towards large volume disease and the possibility of rapid clinical progression. It may have been problematic including patients in this study with small volume CNS disease, however, as many clinicians might adopt an expectant policy in the absence of symptoms in this situation. A further potential criticism of this study is that O^6^-alkylguanine transferase levels were not measured: this would have been of interest despite the lack of activity of temozolomide and lomustine, although this is a difficult patient group to obtain serial tissue samples, given the frequency of rapidly progressive disease.

Temozolomide remains under evaluation in a variety of settings in phase II trials for the treatment of brain metastases from melanoma. One strategy has been to combine temozolomide with other agents; for example, the combination of extended schedule temozolomide and thalidomide (Hwu *et al*, 2005) can be administered safely and results in responses in just over 10% of patients. A second strategy has been to combine temozolomide with whole brain irradiation, the standard treatment for melanoma metastatic to the brain (Margolin *et al*, 2002; Hofmann *et al*, 2006). The administration of temozolomide according to both the standard (Hofmann *et al*, 2006) and an extended (Margolin *et al*, 2002) schedule in combination with whole brain irradiation is generally safe and well-tolerated, but results in responses in only about 10% of patients.

In conclusion, the MTD of temozolomide and lomustine in patients with brain metastases from melanoma is temozolomide 150 mg m^-2^ given on days 1–5 every 28 days and lomustine 60 mg m^-2^ given on day 5 every 56 days. This dose causes significant myelosuppression in terms of thrombocytopaenia and neutropaenia in approximately one-third of patients and does not demonstrate activity in this setting. The further evaluation of the combination of temozolomide and lomustine for the treatment of brain metastases from melanoma therefore is not warranted.

## Figures and Tables

**Table 1:** Dose escalation regime for temozolomide and lomustine

	**Temozolomide dose (mg m^−2^)**	**Lomustine dose (mg m^−2^)**
**Dose level**	**Days 1 to 5 p.o. q 28 days**	**Day 5 p.o. q 56 days**
1	100	60
2	150	60
3	150	80

**Table 2:** Baseline patient characteristics

	***n***	**%**
Sex		
Male	14	54
Female	12	46
		
*Age/years*		
Median (range)	50 (27–63)	
		
*Time from diagnosis of melanoma to brain metastases/(months)*		
Median (range)	36 (1–252)	
		
*Time from diagnosis of brain metastases to trial entry/(months)*		
Median (range)	1 (1–8)	
		
*Prior therapies*		
Adjuvant immunotherapy	7	27
Radiosurgery	1	4
Resection of brain metastasis	1	4
None	17	65
		
*Performance status*		
Median (range)	1 (0–2)	

**Table 3:** Number of cycles of treatment administered at recommended Phase II dose (dose level 2, *n*=20 patients)

**Number of cycles administered**	***n***	**%**
1	13	65
2	5	25
3	1	5
4	—	—
5	—	—
6	1	5

**Table 4:** Outcomes of therapy at recommended Phase II dose (dose level 2, *n*=20 patients)

**Outcome**	***n***	**%**
Response to therapy	0	0
Completed six cycles of therapy	1	5
Progressive disease in brain	10	50
Progressive disease outside brain	3	15
Toxicity necessitating withdrawal from trial	6	30

**Table 5:** Toxicity at recommended Phase II dose (dose level 2, *n*=20 patients)

	**All grades**	**Grade 3–4**
	***n* (%)**	***n* (%)**
*Haematological*		
Thrombocytopaenia	11 (55)	7 (35)
Leucopaenia	6 (30)	6 (30)
Neutropaenia	6 (30)	6 (30)
Anaemia	5 (25)	2 (10)
		
*Nonhaematological*		
Fatigue	10 (50)	— (−)
Nausea	7 (35)	1 (5)
Constipation	6 (30)	1 (5)
Headache	5 (25)	— (−)
Haemorrhage	5 (25)	4 (20)
Pain	2 (10)	— (−)
Dyspnoea	2 (10)	1 (5)
Sepsis	2 (10)	2 (10)
Diarrhoea	1 (5)	— (−)
Pulmonary fibrosis	1 (5)	1 (5)
